# Monthly Summary

**Published:** 1871-06

**Authors:** 


### MONTHLY SUMMARY.

*Effect of Inhalation of Oxygen upon the Pulse.*—Dr. Andrew H. Smith recently read a paper before the N. Y. Medical Journal Association (*Medical Record*, Jan. 2, 1871, p. 481), giving interesting results of experiments with oxygen, eight gallons of which, on the average were administered to each person. The conclusion arrived at was that this gas is an arterial, or more correctly, a cardiac, sedative; the first step being a change in the blood, which facilitates its circulation and thus lessens the labor imposed upon the heart.

In 102 observations made upon 11 phthisical patients, the pulse was retarded an average of 10 beats per minute in 72 of these, was unchanged in frequency in 18, and slightly accelerated in 13. In 12 healthy individuals selected for experiment, in 8 there was an average fall of 9 beats, while in the other 4 there was no change. The sphygmopraph indicated a change also in the character of the beat,—the volume of the pulse being increased, probably on account of a greater time being afforded, by the retardation of the pulse, for the ventricles to become filled with blood, and an increased amount of blood being thus thrown into the arteries. An antagonistic effect of the gas upon the volume of the pulse is found in the fact that it acts upon the blood in such a way as to facilitate its flow through the capillaries, and, as one or the other effect predominates, the pulse becomes increased or diminished in size, or, if they are exactly apportioned to each other, no change of volume results.

Under the use of oxygen, dicrotism of the pulse was usually lessened, owing to the diminution of capillary resistance, and irregularity of the beat was generally overcome, at least temporarily.—*Medical Times.*

---

*Treatment of Snake Bite.*—Dr. J. Fayrer, who has tried more experiments with snake-poison than any other person, and had large experience in the treatment of snake-bites, has published in the *Indian Annals of Medical Science*, December, 1870, an interesting article on this subject, from which we extract the following summary of the treatment he advises.

Apply, at once, a ligature, or ligatures, at intervals of a few inches, as tight as you can possibly tie them ; and the one nearest to the wound tighten, by twisting with a stick or other such agent. Scarify the wound, and let it bleed freely. Apply either a hot iron, or live coal, or explode some gunpowder over the part; or apply either carbolic or some mineral acid or caustic. Let the patient suck the wound while you are getting the cautery ready, or if any one else will run the risk, let him do it.

If it be a positive bite on a toe or finger, especially if the snake have been recognized as a deadly one, either completely excise, or amputate immediately at the next joint. If the bite be on another part, where a ligature cannot be applied, or indeed if it be on the limbs above the toes or fingers, cut the part out at once, completely.

Let the patient be quiet. Do not fatigue him by exertion. When, or even before symptoms of poisoning make their appearance, give eau-de-luce, or liq., or carbonate of ammoniæ, or, even better than these, hot spirits and water. There is no occasion to intoxicate the person, but give it freely, and at frequent intervals.

If he become low, apply sinapisms and hot bottles, galvanism or electro-magnetism, over the heart and diaphragm. Cold douches may also be useful.

The antidotes, in addition, may be used by those who have faith in them; but, as I have said, I fear there is no reason to believe that they are of any use. Encourage and cheer the patient as much as possible.

As to local effects, if there be great pain, anodynes may be applied or administered, and antiseptic poultices to remove sloughs; collections of matter must be opened.

Other symptoms to be treated on general surgical principles.—*American Journal of the Medical Sciences.*

---

*Mustard to Make Leeches Take.*—"Having had occasion to order a mustard poultice for a patient, it became requisite to put some leeches on the same place. I was told that they fastened instantly, filled rapidly, and that the blood streamed afterwards into bread poultices as if it would never stop. I took the hint; and now, whenever I order leeches, I always have a mustard poultice applied first, then the leeches (two or three, instead of half a dozen,) and then bread poultices. There is less trouble for those who have to apply the leeches, far less annoyance, weariness, and exhaustion for the patient, and a much more satisfactory result. The flow of blood is, however, sometimes so much greater than would be thought likely or possible that I think it right to add a few words of caution. A few days ago, one of my patients, a young lady grown up, and of average strength, bled to fainting from only two leeches applied in this way.—*R. L., London Lancet.*

---

*A New Anœsthetic.*—Dr. Liebreich, of Berlin, asserts that he has discovered a substitute for chloroform, the use of which is free from all the disagreeable sensations consequent upon the use of that drug. He calls it æthyliden chloride. It is a colorless fluid, of an agreeable odor and very volatile. Sleep suddenly overtakes the inhaler, and he wakens quickly and involuntarily, as from a natural slumber.

If this statement be true, Dr. Liebreich will prove himself one of the greatest benefactors of the human race that has lived. It were glory enough for one man to have introduced so great a boon as chloral hyrate.—*Med. and Surg. Rep.*

---

*Earth Eating.*—It is well known that, in different parts of the world, there are people who eat earth; among them are some of the natives of Java, who eat a red kind of earth as a luxury. This earth, which is soft and smooth to the touch, has been analyzed by a German chemist, who finds it very rich in iron, with a small quantity of potassa and soda. Some tribes eat earth to stay the pangs of hunger by filling their stomachs, and because at times they can get nothing better ; but the people in Java eat their earth, baked in thin cakes, as an agreeable variety in their general diet. The cakes when slightly moistened, are rich and unctuous, and the enjoyment in eating is supposed to consist in the sensation produced by a fatty substance. It is a curious fact in the history of human habits.— *Virginia Clinical Record.*

---

*Spontaneous Combustion of the Human Body.*—Mr. A. B. Flowers, of Alexander, La., writes us that the statement made in a recent article on this subject, to the effect that no one has ever witnessed a case of spontaneous combustion in the human body, is a mistake, as he was himself with several others, an eye-witness to a case of the kind. The person who was the victim was a hard drinker, and was sitting by a fire, surrounded by Christmas guests, when suddenly flames of a bluish tint gushed from his mouth, and he was soon a corpse. The body, he states, remained extremely warm for a much longer period than usual.—*Scientific American.*

---

*Safety of Hyperdermic Injections.*—Dr. Charles Hunter, who was largely instrumental in introducing the use of the hyperdermic syringe, declared lately, in the London Clinical Society, that, speaking from an experience of nearly two thousand cases annually, he saw no risk in the system of hyperdermic injection, whither there was evidence of heart disease or not. He adds, "Care should be used at the first injection in looking to the state of the kidneys. It was possible that tetanus might result from puncturing a nerve, but he had never seen it."—*Pacific Medical and Surgical Journal.*

---

*Treatment of Enlarged Tonsils in Children.*—Dr. James Martin states that an eminent Dublin practitioner finds the sulphate of potassa, administered daily for a month or six weeks, almost a specific for enlarged tonsils in children. From five to fifteen grains are given every morning with a small quantity of rhubarb and aromatics. The dose should produce mere laxity of the bowels, and must be diminished if it causes purging.—*Druggist's Circular.*

*A Pleasant Remedy for Sea-Sickness.*—There have been many suggestions made as to the prevention of sea-sickness, none of which have, to say the least, been found completely successful in practice. The introduction into practice of hydrate of chloral, which produces with certainty sleep for a definite number of hours, has suggested the means of escaping the horrors of a short sea passage at least, and possibly of mitigating the most prolonged horrors of sea-sickness. To go asleep at Dover, and wake to find one's self Calais, is a plan which, failing other expedients, has in it much promise. An ordinary dose of hydrate of chloral produces sleep usually in a quarter of an hour, and with almost unfailing certainty. Some cases just published by Dr. Doring, of Vienna, seems to show that the value of hydrate of chloral to obviate sea-sickness is very great, It produces quiet and prolonged sleep. In all the instances recorded, it seems to have been of great value, even during prolonged sea voyages, giving good night's rest, arresting violent sickness when it had set in, and stopping the tendency to its recurrence.—*British Medical Journal.*

---

*New Plastic Material.*—A beautiful plastic substance which the inventor has given the somewhat pretentious name of artificial ivory, can be prepared by mixing collodion with phosphate of lime. The phosphate should be pure, or the color of the compound will be unsatisfactory. On setting, the mass is found to be hard and susceptible of a very fine polish. The material can be used extensively, applied in modes that will suggest themselves to any intelligent artist, to high class decoration.—*Dental Advertiser.*

---

*Cheap Retort.*—A writer in the *British Journal of Dental Science*, says: "When first we commenced to make gas, we used the large glass flasks sent with the apparatus, costing about three shillings apiece. A box full of broken glass testifies to the persevverance and constant mortification under this regimen. All kinds of flasks and retorts, stoppered and plain, were tried, but all proved fragile. Now we use a common stone-ware gallon beer bottle, price four pence; put in the nitrate, and when thoroughly warmed and hot, by raising the heat slowly we find a cheap substitute."

*Ether Spray in Ulcers and Cutaneous Affections.*—M. Peuch, a distinguished French vetinary surgeon, quoted by the *Lyon Medical* from the *Jou.. de Med. Vet de Lyon*, has used the ether spray with marked success in ulcers and divers cutaneous affectins in the lower animals. Crusts, where they exist, are detached by the retraction of the subjacent tissues, the distressing itching of certain lesions is at once allayed, and an exposed surface is dried by the rapid evaporation without the irritation caused by the atmospheric air. Complete congelation is not sought, and in the case of said surfaces, the spray is used only until the deep red of the tissues is reduced to a pale pink. Under these conditions, M. Peuch asserts that spray is a most valuable cicatrizing agent in wounds and ulcers, more especially those of an indolent nature. The experiment may be worth a trial in the human subject, as we have no very satisfactory, means of either allaying itching or healing chronic ulcers.—*Naitonal Medical Journal.*

---

*Sulphur in Croup.*—Dr. Lanini, in *Lo Sperimentale*, of Florence, of December, 1870, writes that he has treated membranous croup successfully with powdered sulphur, in doses of a scruple every two hours. He reports a case occurring in a girl of eight years, where all other remedies with which he was acquainted had failed to give relief. After the second dose of the sulphur, the dry cough was diminished, and she soon began to expectorate casts of the bronchial tubes, some of which were nearly an inch in length. The treatment was continued two days, and the patient did well. The doctor was induced to try the remedy in consequence of the experiments and recommendations of Dr. Banieri Bellini, Professor of Toxicology in the Royal Institute at Florence, published in the September number of the *Sperimentale* of 1869.—*Med. Record.*

---

*Creasote in Mercurial Salivation.*—This is a very old but very efficieut remedy: Caeasote ʒ ss; Sage Tea, one pint, to be used as a gargle every hour, for one or two days. Keep the bowels soluble by the use of saline aperients.—*Oregon Medical and Surgical Reporter.*

*Maggots in the Ear.*—Dr. C. Robertson, of Albany, N. Y. (*Buffalo Med. and Surg. Jour*), at the December meeting of the Albany County Medical Society, spoke of the case of a lady, who, while on a pic-nic, heard a fly buzzing about her ears, but did not think of the circumstance again until after the lapse of a few days, when she felt some irritation in one ear. When consulted, Dr. R—— removed some parasites with aural forceps, which had penetrated beyond the membrana tympani. Sweet oil was poured into the ear, which was retained for awhile; shortly, one came to the surface, apparently searching for breath; this gave relief for ten minutes. More were observed, which were extracted with forceps. The after-treatment consisted in syringing the ear with warm water. The opening of the tympanum closed, and her hearing became perfect.

---

*Abstinence Extraordinary.*—There are few things too miraculous for the simple medical faith of the rural press; and the latest evidence of credulity in this respect is a recital of the case of a reverend gentleman in Bennington, Vermont, who, under the advice of his physicians, abstained wholly from food for thirty-five days, since the expiration of which fast his entire sustenance has been one ounce of biscuit and two ounces of apple daily. The tendency of the clergy to commit suicide or homicide with therapeutic intent, is well known to us; but, irrespective of the impossibility of surviving such a fast, it is quite beyond belief that any "physicians" should recommend starvation as a remedial agent.—*Med. Gazette.*

---

*The Title of "Doctor."*—"The title of 'Doctor' was invented in the twelfth century. Irnerius, a learned professor of law at the University of Bologna, induced the emperor Lothaire II., whose chancellor he was, to create the title, and he himself was the recipient of it. He was made doctor of laws by that university. Subsequently the title was borrowed by the faculty of theology, and first conferred by the University of Paris on Peter Lombard. William Gordenio was the first person upon whom the title of doctor of medicine was bestowed; he received it from the College of Asti, in 1329."—*Medical Gazette.*

